# Genetic insights into dietary patterns, liposome mediation, and osteoporosis risk: a Mendelian randomization study

**DOI:** 10.3389/fnut.2024.1389896

**Published:** 2024-10-03

**Authors:** Kehan Long, Tengfei Zheng, Ao Gong, Zhendong Ying, Lei Zhang

**Affiliations:** ^1^Department of Orthopedics, The Third Hospital of Mianyang-Sichuan Mental Health Center, Mianyang, China; ^2^Department of Orthopedic Surgery, The First Affiliated Hospital of Shandong First Medical University & Shandong Provincial Qianfoshan Hospital, Shandong Key Laboratory of Rheumatic Disease and Translational Medicine, Jinan, Shandong, China; ^3^Second Clinical Medical College of Shandong University of Traditional Chinese Medicine, Jinan, Shandong, China; ^4^The First Affiliated Hospital of Shandong First Medical University, Shandong Provincial Qianfoshan Hospital, Jinan, Shandong, China

**Keywords:** osteoporosis, dietary habits, liposomes, Mendelian randomization, GWAS, causal inference

## Abstract

**Background:**

This study examines the indirect causal relationships between dietary habits and osteoporosis, mediated through liposomes, utilizing a two-sample Mendelian randomization (MR) approach. The research leverages genetic variations as instrumental variables to explore the genetic influences on dietary habits, liposomes, and osteoporosis, aiming to unravel the complex interplay between diet, lipid metabolism, and bone health.

**Methods:**

The study utilized genome-wide association studies (GWAS) data for liposomes from Finnish individuals and osteoporosis-related data, alongside dietary factors from the OpenGWAS database. Instrumental variables were selected based on genetic variants associated with these factors, using a strict significance level and linkage disequilibrium threshold. Statistical analysis employed the Inverse Variance Weighted method, weighted median, and mode-based methods within the R environment, complemented by sensitivity analyses to ensure the robustness of the causal inferences.

**Results:**

Findings revealed significant causal relationships between specific dietary components (white rice, cereal, and non-oily fish) and osteoporosis risk, both directly and mediated through changes in liposome levels. Notably, white rice consumption was associated with an increased risk of osteoporosis, while cereal and non-oily fish intake showed protective effects. Further, certain liposomes were identified as mediators in these relationships, suggesting a link between diet, lipid profiles, and bone health.

**Conclusion:**

The study highlights the significant impact of dietary habits on osteoporosis risk, mediated through liposomes. These findings underscore the importance of considering lipidomic profiles in dietary guidance and suggest potential targets for preventing osteoporosis through nutritional interventions.

## 1 Introduction

Osteoporosis, characterized by reduced bone density and heightened fracture risk, exerts a substantial public health burden worldwide (1). Dietary and lifestyle factors strongly influence osteoporosis development and progression (2, 3). Elucidating nutritional influences on bone metabolism could inform preventive strategies targeting modifiable risk factors. However, conventional observational studies are limited in assessing causal diet-disease effects due to residual confounding and reverse causation. Mendelian randomization (MR) analysis utilizes genetic variants to strengthen causal inference between modifiable exposures and disease outcomes (4). Here, we applied two-sample MR to evaluate potential causal effects of diverse dietary components on osteoporosis risk, and further investigated the mediating role of plasma lipids called liposomes in these diet-osteoporosis associations.

Bone metabolism reflects a delicate balance between bone formation by osteoblasts and bone resorption by osteoclasts. Imbalances tilting toward excessive resorption underlie osteoporosis pathogenesis (5, 6). Nutrient status and bioactive food compounds can modulate cellular processes influencing skeletal integrity. For instance, calcium and vitamin D are essential for bone mineralization (7). Protein intake promotes insulin-like growth factor-1 production, stimulating osteoblast proliferation (8). Plant phytochemicals may exert anti-inflammatory and antioxidant effects beneficial for bone health (9). However, most diet-osteoporosis studies rely on error-prone food frequency questionnaires with findings often confounded by linked lifestyle factors. Establishing causal effects requires alternative study designs less susceptible to reverse causation and confounding.

MR leverages genetic variants associated with modifiable exposures to strengthen causal inference. Since genotypes are randomly allocated at conception, MR is not prone to confounding by environmental factors (10). Further, germline genetic variants cannot be influenced by disease processes, avoiding reverse causation. Two-sample MR utilizes summary-level data from large genome-wide association studies (GWAS), enhancing statistical power to detect causal effects (11). Here, we applied two-sample MR using GWAS data on osteoporosis and dietary factors alongside genetic instruments for plasma liposomes, major transporters and structural components influencing metabolic pathways. Examining liposomes as potential mediators can provide biological insight into diet-osteoporosis mechanisms.

Plasma liposomes encompass a diverse range of lipids including phosphatidylcholines, sphingomyelins, and triglycerides. As core constituents of cell membranes and lipid rafts, liposomes impact signaling related to bone turnover (12). Lipid compositional changes also reflect systemic metabolic aberrations affecting bone. For instance, hyperlipidemia induces reactive oxygen species and inflammatory cytokines that stimulate osteoclastogenesis (13). Evaluating liposomes as intermediates in diet-osteoporosis relationships can thus elucidate relevant lipid pathways.

This study applied stringent criteria for selecting genetic variants associated with each exposure meeting genome-wide significance. Analyses were conducted using robust MR methods including inverse-variance weighted (IVW), weighted median, and mode-based estimation. Sensitivity analyses evaluated assumption violations. We first assessed causal relationships between dietary factors and osteoporosis risk. Next, we determined causal effects of diet on liposome levels. Finally, we examined causal associations between liposomes and osteoporosis, identifying potential mediators.

Findings from this MR study nominate promising dietary targets for osteoporosis prevention and highlight lipidomic pathways mediating nutritional effects on bone. Results will guide future mechanistic studies on diet-induced metabolic shifts influencing osteoblast-osteoclast homeostasis. From a clinical perspective, delineating causal risk factors enables evidence-based dietary recommendations for at-risk groups. For instance, identifying foods causally linked to lower osteoporosis odds could inform dietary guidelines for postmenopausal women. Overall, applying genetic epidemiology tools to integrate multi-omics datasets represents a powerful approach to unravel the complex interplay among diet, metabolism, and disease. Findings will be translated through cross-disciplinary collaboration with nutrition scientists and bone biologists, working toward reducing the rising global burden of osteoporosis.

## 2 Materials and methods

### 2.1 Study design

Utilizing a dual-sample MR approach, this study aims to assess the indirect causal relationship between dietary habits and osteoporosis, mediated by the role of liposomes. MR leverages genetic variations as proxies for risk factors, making it crucial for chosen instrumental variables (IVs) to adhere to three essential criteria for accurate causal inference: (1) The genetic variation must have a direct link to the factor of exposure; (2) There should be no association between the genetic variation and any potential confounding variables that could affect both the exposure and the outcome; (3) The genetic variation's impact on the outcome must exclusively proceed through the exposure, without any alternative routes (14–16).

### 2.2 Obtaining all GWAS data

The detailed GWAS (Genome-Wide Association Studies) summary data pertaining to each liposome is available for public access in the GWAS Catalog, listed under registration numbers from GCST90277238 to GCST90277416. This document details both univariate and multivariate GWAS analyses covering 179 lipid classes across 13 lipid categories, conducted on a sample of 7,174 Finnish individuals from the GeneRISK study cohort (17). Following this, a comprehensive Phenome-Wide Association Study (PheWAS) was carried out on lipid-associated genetic loci, involving 377,277 participants from a biobank (18). Additionally, co-localization analysis was applied to these findings. The osteoporosis-related data was sourced from the Finnish R10 version, which includes data on 8,017 cases and 391,037 controls (19). Information on dietary factors was obtained from the OpenGWAS database (20).

### 2.3 Selection of instrumental variables

In the selection of IVs, specific significance levels and Link Unbalance (LD) thresholds were set to ensure a sufficient association between the chosen genetic variants and the exposure factors studied (such as liposome levels, osteoporosis, and dietary factors) while minimizing potential confounding influences.

For each liposome, the significance level was set at 1 × 10^−5^. This stringent threshold is used to select genetic variants directly associated with liposome levels from a vast number of gene loci (21). For osteoporosis studies, the significance level was adjusted to 5 × 10^−8^, a commonly used standard for genome-wide significance in GWAS, ensuring that the selected IVs are statistically robust and can stand out in genome-wide association studies (22). For the selection of IVs related to dietary factors, except for specific dietary intakes such as shellfish, lamb, whole eggs, sweets, mixed vegetables, white rice, and dark chocolate intake, which have a significance level of 5 × 10^−6^, all other dietary factors were set at 5 × 10^−8^.

When selecting IVs, we used the “TwoSampleMR” data packet, setting the LD threshold at *R*^2^ < 0.001 and an aggregation distance of 10,000 kb (23). This setup helps in selecting genetic loci that are independent at lower linkage disequilibrium levels, avoiding estimation biases caused by high linkage disequilibrium among genetic variants in the same area. The low LD threshold ensures that the chosen genetic variants are statistically independent, avoiding errors introduced by multiple genetic variants in the same region affecting the results simultaneously. We calculate the F value of a single SNP and exclude SNPs with F < 10, and finally exclude palindrome SNPs (24).

### 2.4 Statistical analysis

Dual-sample MR analysis of positive metabolites on immune cell phenotypes was conducted using the R software, version 4.2.1. This software represents a comprehensive statistical computation and graphics environment, available at R Project website (http://www.Rproject.org) (25). The analysis was facilitated by the “TwoSampleMR” package (version 0.5.7) within the R environment, which is specifically tailored for MR analysis. This package offers a suite of tools for the estimation, testing, and conducting sensitivity analyses of causal effects. The Inverse Variance Weighted (IVW) method, a cornerstone technique in MR, integrates Wald estimates from various genetic variations (the ratio of SNP-outcome associations to SNP-exposure associations), employing the inverse variance of each SNP outcome for weighting (26). Weighted median and mode-based methods are also utilized as auxiliary approaches to furnish robust causal inferences, even when some instrumental variables might be invalid, provided certain prerequisites are met (27). The integrity of these analyses is further bolstered by exhaustive sensitivity analyses, including Cochran's *Q*-test, to scrutinize the heterogeneity amongst instrumental variables (28). This meticulous statistical scrutiny guarantees that the findings are as dependable and precise as achievable, based on the dataset.

### 2.5 Mendelian mediation analysis

Initially, the study focuses on discerning the causal link between dietary factors and osteoporosis, aiming to negate the impact of any reverse causality in this phase. The investigation then progresses to examine the causal influence of liposomes on osteoporosis. From the dietary factors, three positive influences are selected for further analysis in conjunction with liposomes to pinpoint the mediating agents. The overall effect of dietary factors on osteoporosis is broken down into direct and indirect impacts. The indirect effect represents the product of the causal relationship between dietary factors and liposomes (denoted as β value) and the causal relationship between liposomes and osteoporosis (also denoted as β value) (29). The direct effect is determined by subtracting the indirect effect from the total effect. The ratio of mediation is calculated by dividing the mediation effect by the total effect, providing a quantitative measure of the mediation's contribution to the total effect (30).

## 3 Results

### 3.1 The causal relationship between dietary factors and osteoporosis

**Figure 1:**
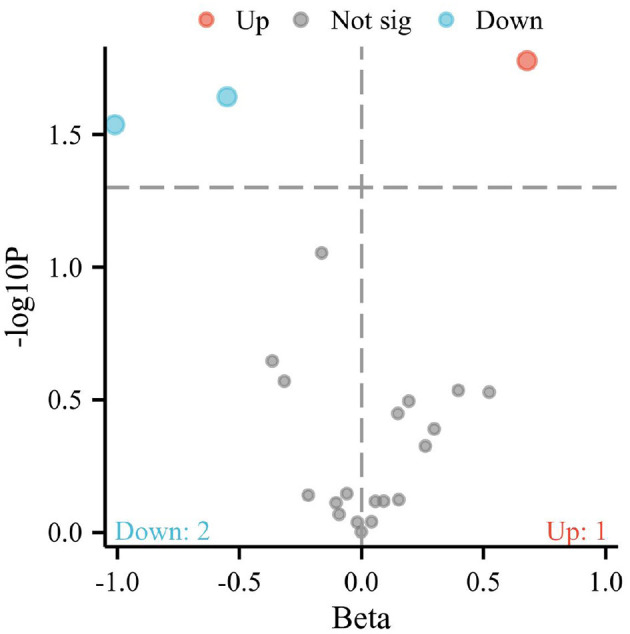
Volcano plot representing the causal effects of dietary factors on the risk of osteoporosis. The x-axis (Beta) displays the effect size of each dietary component on osteoporosis risk, while the y-axis (–log10 p) reflects the statistical significance of each association. Points above the horizontal threshold line indicate a statistically significant relationship. Colored points represent the direction of the association: red for an increase (Up), blue for a decrease (Down) in the risk of osteoporosis, and gray for non-significant (Not sig) results.

**Figure 2:**
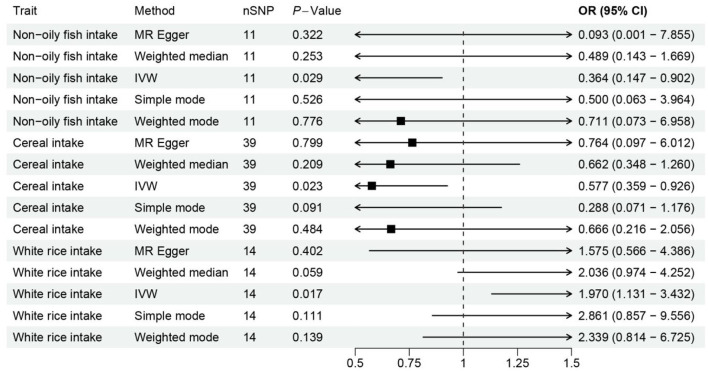
Forest plot visualizing the Mendelian randomization analysis of the causal relationship between the three dietary factors and the risk of osteoporosis. The plot displays the odds ratios (ORs) and their 95% confidence intervals (CIs) for each dietary factor, estimated using multiple Mendelian randomization methods such as Weighted mode, Weighted median, Simple mode, MR Egger, and Inverse variance weighted, represented by different colored symbols. Points to the right of the vertical line (OR = 1) indicate an increased risk of osteoporosis, while points to the left suggest a potential protective effect against the disease.

The IVW method, an analytical technique commonly utilized in Mendelian randomization studies, has been instrumental in elucidating the causal relationships between dietary factors and the risk of developing osteoporosis. The significance of this approach lies in its ability to leverage genetic variants as instrumental variables, thus providing a more robust inference on causality by mitigating confounding factors typically encountered in observational studies. In a recent analysis, conducted at a significance level of 0.05, the IVW method has illuminated intriguing associations between specific dietary components and osteoporosis risk, as depicted in Figures 1, 2.

Notably, the intake of white rice was found to exhibit a positive correlation with osteoporosis, with a *P*-value of 0.016, indicating a statistically significant association. The effect size (β) of 0.677 translates to an Odds Ratio (OR) of 1.969, with a 95% confidence intervals (CI) ranging from 1.130 to 3.431. This finding suggests that higher consumption of white rice may increase the risk of developing osteoporosis, highlighting a potential dietary risk factor that warrants further attention in nutritional guidance and public health policies.

Conversely, the intake of cereal and non-oil fish demonstrated a protective association against osteoporosis. Cereal consumption was associated with a *P*-value of 0.022, a β of −0.550, an OR of 0.576, and a 95% CI of 0.359–0.926. Similarly, non-oil fish intake showed a *P*-value of 0.029, a β of −1.010, an OR of 0.364, and a 95% CI of 0.147–0.901. Non-oily fish intake has been associated with a protective effect against osteoporosis, mediated by its impact on lipid profiles, particularly phosphatidylcholines and other beneficial lipids that influence bone health. Omega-3 fatty acids present in non-oily fish, such as DHA and EPA, are known to suppress osteoclastogenesis and maintain skeletal integrity by producing anti-inflammatory eicosanoids (31, 32). These findings indicate that increased intake of cereals and non-oil fish is negatively correlated with osteoporosis risk, suggesting a protective effect that could be beneficial in dietary strategies aimed at reducing osteoporosis prevalence.

### 3.2 The causal relationship between three positive dietary factors and liposomes

The utilization of the IVW method, set against a significance level of 0.05, has facilitated a deeper investigation into the causal relationships between dietary factors and the presence of liposomes, a type of lipid molecule, within the body. In this comprehensive analysis, three dietary components were meticulously examined for their causal associations with various types of liposomes, revealing a complex interplay between diet and lipid profiles.

The analysis unveiled a positive causal relationship between the intake of white rice and the levels of seven distinct types of liposomes. This finding, detailed in Supplementary Material 1, suggests that an increased intake of white rice may elevate the levels of certain liposomes within the body. Higher consumption of white rice has been linked to an increased risk of developing osteoporosis. This association is suggested to be due to its impact on lipid profiles, specifically increasing certain types of liposomes that may negatively affect bone metabolism (33). The nature of these liposomes and their implications on health necessitate further exploration to understand the potential metabolic and systemic effects associated with white rice consumption.

Remarkably, the intake of non-oily fish was found to have a positive causal relationship with 14 types of liposomes, as outlined in Supplementary Material 2. This result indicates that consuming non-oily fish contributes to higher levels of a broader range of liposomes compared to white rice intake. Given the general perception of fish as a healthy dietary component, especially in the context of cardiovascular health, these findings prompt a reevaluation of how non-oily fish affects lipid metabolism and what these specific liposomes signify in terms of health outcomes.

**Figure 3:**
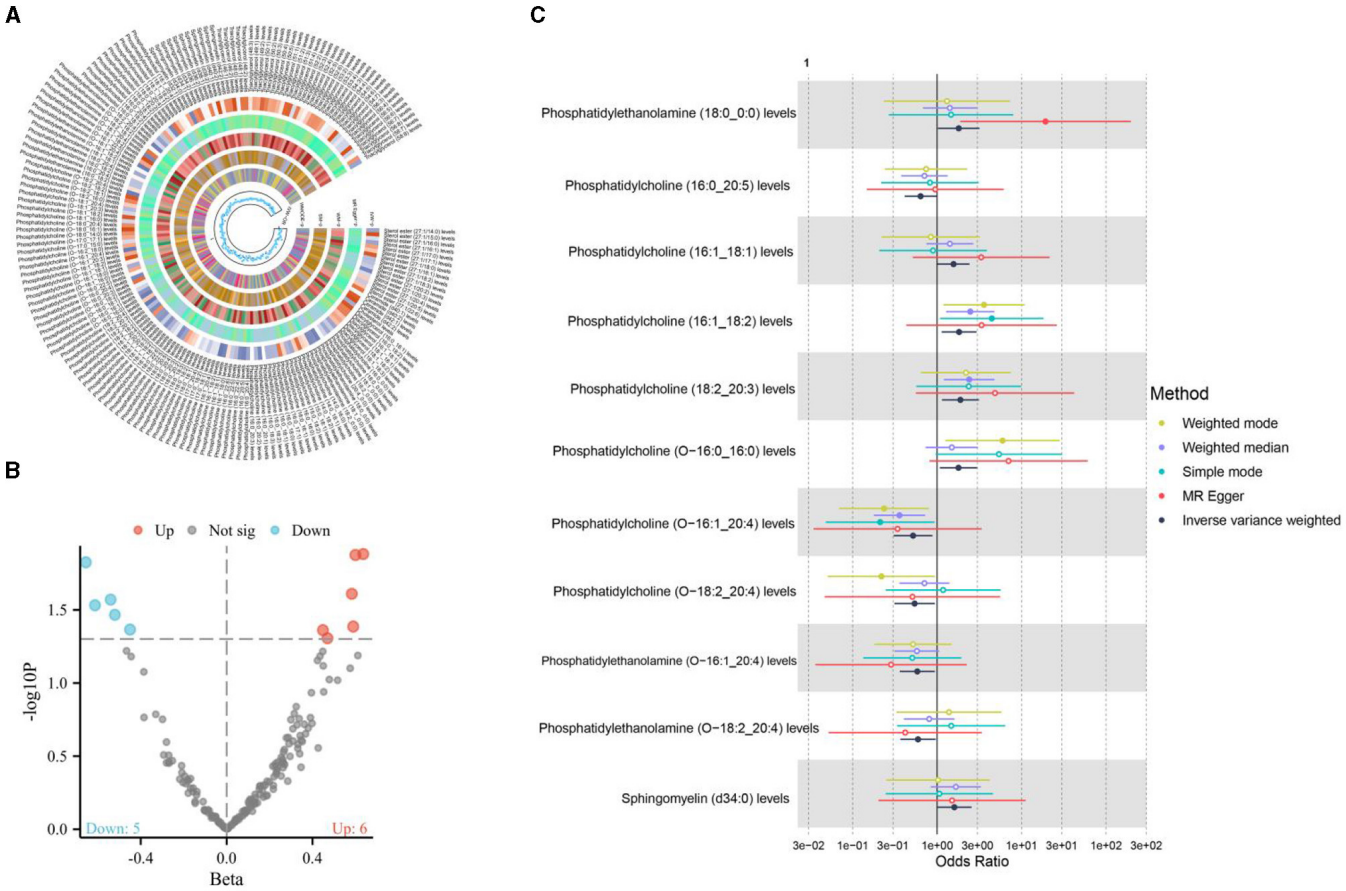
**(A, B)** The causal relationship between cereal intake and liposomes; **(C)** The causal relationship between cereal intake and 11 positive liposomes.

Cereal intake demonstrated a positive causal association with 11 types of liposomes, as shown in Figure 3. Cereal intake has been shown to have a protective effect against osteoporosis. This is partly mediated by increased levels of phosphatidylcholine containing linoleic and arachidonic acids, which modulate eicosanoid balance favoring bone formation over resorption. Additionally, cereals contribute to bone health through their fiber, micronutrients (like magnesium and zinc), and phytochemicals, which likely enhance the synthesis of beneficial lipids and support bone metabolism (33, 34). This suggests that cereals, a staple in many diets and often considered a healthy food choice due to their fiber content and nutrient profile, can influence the body's lipid composition in significant ways. The specific types of liposomes associated with cereal intake and their potential roles in health and disease offer a fertile ground for further investigation.

### 3.3 The causal relationship between liposomes and osteoporosis

**Figure 4:**
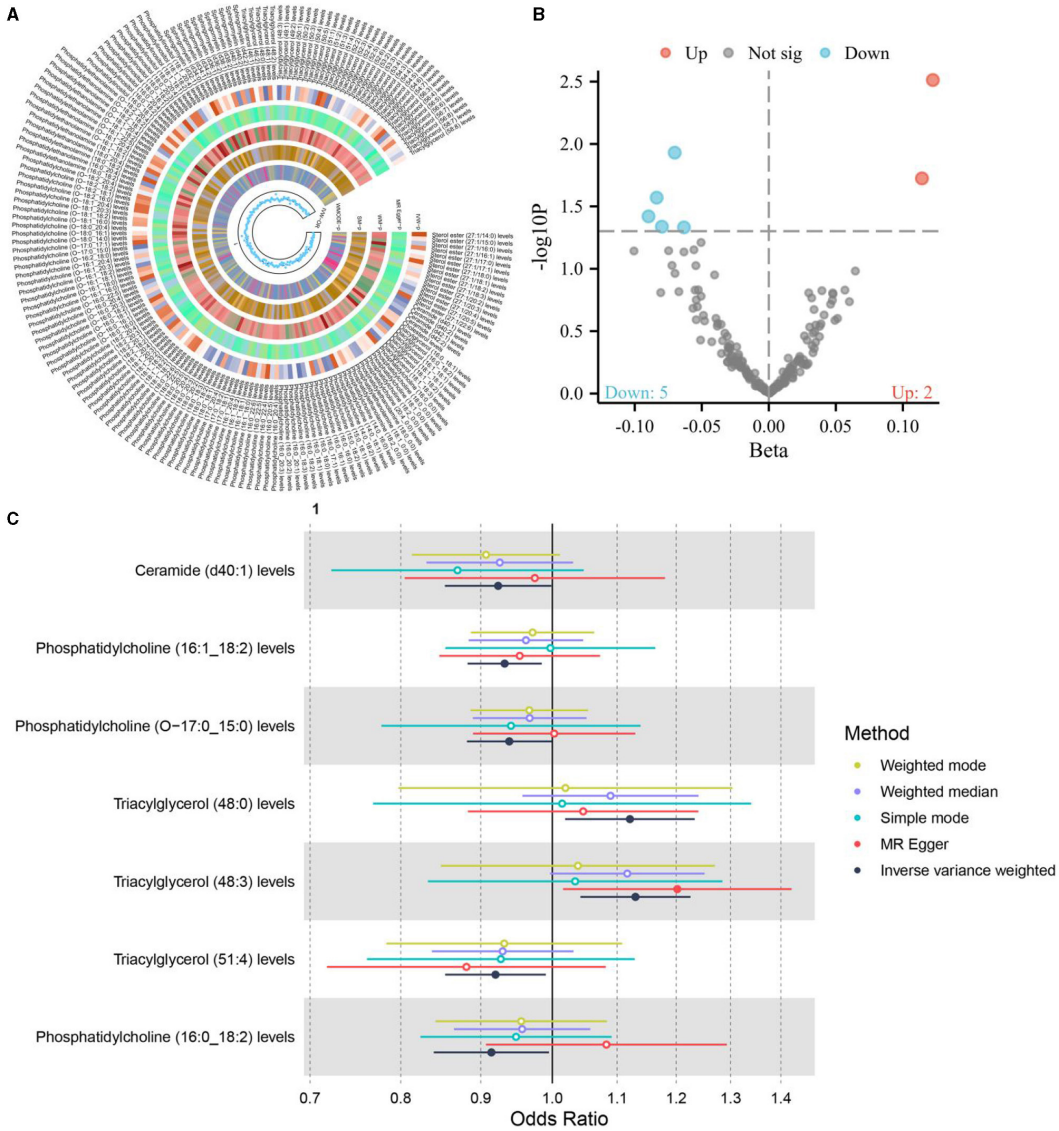
**(A, B)** The causal relationship between liposomes and osteoporosis; **(C)** The causal relationship between 11 positive liposomes and osteoporosis.

The application of the IVW method, with a significance level set at 0.05, has provided insightful revelations into the intricate connections between liposomes and osteoporosis, a common bone disorder characterized by decreased bone density and increased fracture risk. In an exhaustive analysis of 179 liposomes, the research identified a positive causal relationship between seven specific liposomes and osteoporosis, as illustrated in Figure 4. This finding significantly advances our understanding of the lipidomic influences on bone health and disease, suggesting that alterations in the levels of certain liposomes could influence the pathogenesis or progression of osteoporosis. The identification of these liposomes opens new avenues for research into their roles in bone metabolism and the potential mechanisms by which they contribute to bone density reduction.

Further analysis of the dietary factors previously identified to have causal relationships with liposomes revealed a nuanced interplay between diet and osteoporosis risk mediated through lipidomic pathways. Among the dietary factors examined—white rice intake, non-oily fish intake, and cereal intake—only cereal intake was found to influence the levels of one of the seven liposomes positively associated with osteoporosis. Specifically, Phosphatidylcholine (16:1_18:2) levels were identified as an intermediary factor linking cereal intake with osteoporosis, suggesting a complex pathway through which diet can modulate disease risk via lipidomic profiles.

This association between cereal intake, Phosphatidylcholine (16:1_18:2) levels, and osteoporosis underscores the importance of considering lipidomic profiles in the context of dietary influences on bone health. Phosphatidylcholine, a major component of biological membranes, plays a crucial role in cellular processes and metabolic pathways. Its identification as a mediator in the relationship between cereal intake and osteoporosis risk highlights the potential for dietary strategies to influence bone health outcomes through modifications in lipidomic profiles.

### 3.4 Reliability evaluation results

The methodological rigor of the study, employing the IVW method within a random effects model, adeptly addresses the complexities inherent in discerning the causal relationships between dietary factors and osteoporosis. The random effects model's allowance for heterogeneity among IVs effectively accommodates the diverse genetic architectures influencing these relationships, rendering the potential variability across studies a non-disruptive element in the analysis.

A crucial step in affirming the validity of the causal inferences drawn from the IVW analysis is the evaluation for horizontal pleiotropy, which occurs when an instrumental variable influences the outcome via pathways other than the exposure of interest. The employment of the MR-Egger intercept test serves as a robust tool for detecting the presence of such pleiotropy. The findings of this test indicated no evidence of horizontal pleiotropy (*P* > 0.05), bolstering the credibility of the causal relationships identified in the study. This absence of pleiotropy suggests that the instrumental variables used in the analysis exert their effects on osteoporosis primarily through the dietary factors under investigation, rather than alternative biological pathways.

Further reinforcing the study's findings, a comprehensive sensitivity analysis was undertaken using the leave-one-out method. This approach, which systematically reevaluates the causal estimate by excluding one instrumental variable at a time, is instrumental in identifying any outliers that might disproportionately influence the analysis results. The absence of outliers in this sensitivity analysis underscores the stability and reliability of the identified associations between dietary factors and osteoporosis, affirming the robustness of the causal inferences.

Lastly, the exploration of a reverse causal relationship, wherein osteoporosis could potentially influence cereal intake, addresses the bidirectional nature of causality that often complicates epidemiological and genetic investigations. The analysis found no evidence of interference from such a reverse causal relationship, as presented in Table 1. This finding further clarifies the directional specificity of the relationship, suggesting that the impact of cereal intake on osteoporosis risk is unlikely to be confounded by reverse causality.

**Table 1:** The reverse causal relationship between osteoporosis and cereal intake.

**Method**	**Nsnp**	**pval**	**or**	**or_lci95**	**or_uci95**
MR Egger	7	0.921	0.990	0.823	1.190
Weighted median	7	0.566	0.993	0.970	1.016
Inverse variance weighted	7	0.674	0.994	0.966	1.022
Simple mode	7	0.253	0.965	0.913	1.019
Weighted mode	7	0.322	0.971	0.922	1.023

### 3.5 Genetic susceptibilities and SNP identification

We utilized GWAS data to identify SNPs associated with dietary factors, liposome levels, and osteoporosis. The genetic variants selected as IVs were those significantly associated with these factors, ensuring robust causal inference. Specifically, SNPs such as rs1296819, rs7412, and rs429358 were identified to be significantly associated with liposome levels and dietary intake. These SNPs were chosen based on their genome-wide significance levels (*p* < 5 × 10^−8^) and linkage disequilibrium thresholds (*R*^2^ < 0.001).

#### 3.5.1 SNPs associated with dietary components

Our findings revealed significant causal relationships between dietary components (such as white rice, cereal, and non-oily fish) and osteoporosis risk. For example, the SNP rs1296819 was associated with white rice intake and showed a significant causal effect on osteoporosis risk with a β value of 0.677 and an OR of 1.969 (95% CI: 1.130–3.431). Similarly, SNPs linked to cereal and non-oily fish intake demonstrated protective effects against osteoporosis, with rs7412 and rs429358 showing significant associations.

#### 3.5.2 Liposome mediation

The mediating role of liposomes was evaluated, identifying specific liposomes like Phosphatidylcholine (16:1_18:2) as significant mediators. Genetic variants such as rs7412 influenced these liposome levels, which in turn affected osteoporosis risk.

### 3.6 Mendelian mediation analysis

The analysis of the mediating effect of liposome Phosphatidylcholine (16:1_18:2) levels on the relationship between cereal intake and osteoporosis provides insightful revelations into the nuanced dynamics governing this association. By decomposing the total effect of cereal intake on osteoporosis into direct and mediating components, the study elucidates the intricate interplay between dietary factors, lipid profiles, and bone health.

The total effect of cereal intake on reducing the risk of osteoporosis was quantified as −0.55, indicating a protective role of cereal consumption against the development of this bone condition. Within this framework, the mediating effect of Phosphatidylcholine (16:1_18:2) levels was calculated to be −0.037. This value represents the portion of the cereal intake effect on osteoporosis that is attributable to changes in the levels of this specific liposome. The direct effect, which quantifies the impact of cereal intake on osteoporosis independent of Phosphatidylcholine (16:1_18:2) levels, was found to be −0.513. This substantial direct effect underscores the significant role of cereal consumption in potentially reducing osteoporosis risk through mechanisms beyond its influence on Phosphatidylcholine (16:1_18:2) levels.

**Figure 5:**
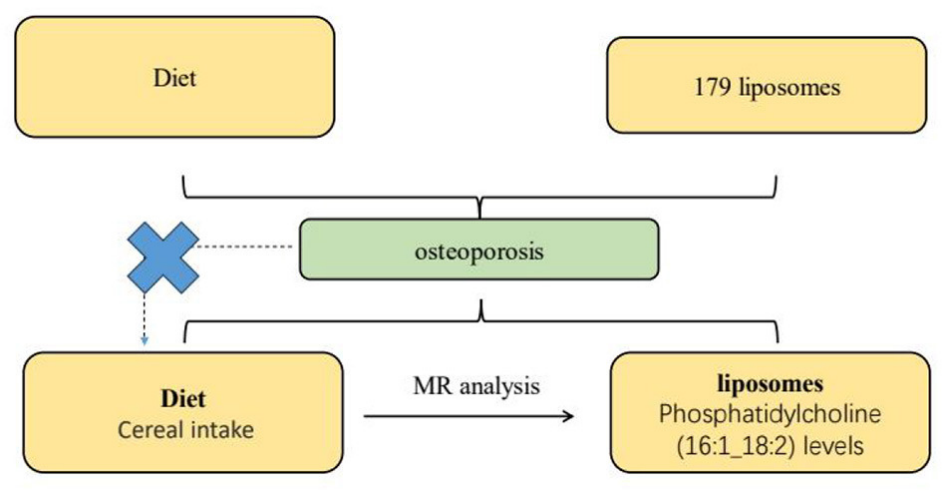
This diagram illustrates the Mendelian randomization (MR) analysis pathway evaluating the potential causal relationship between dietary intake and osteoporosis, mediated by specific lipid components in liposomes. A total of 179 liposome types are selected for the study, with a focus on the levels of liposomes containing Phosphatidylcholine (16:1_18:2), determined through MR analysis. The pathway demonstrates the method used to elucidate the mediating factors in the relationship between diet, lipid composition, and osteoporosis risk.

The mediation rate of 6.9% attributed to Phosphatidylcholine (16:1_18:2) levels reveals that, while the presence of this liposome does play a role in the relationship between cereal intake and osteoporosis, it accounts for a relatively small proportion of the effect. This suggests that the protective effect of cereal intake against osteoporosis is primarily driven by other factors or mechanisms, with Phosphatidylcholine (16:1_18:2) levels serving to slightly attenuate this protective effect. These comprehensive analyses and their implications are visually summarized in Figure 5.

## 4 Discussion

Our dual-sample Mendelian randomization study provides compelling evidence that specific dietary components influence osteoporosis risk through causal effects on plasma liposome levels. These revelations nominate novel targets for nutritional intervention and prognostic biomarker development, while elucidating intricate biological pathways through which diet modulates bone metabolism via impacts on the lipidome.

The finding that the protective effect of cereal intake against osteoporosis is partially mediated by increased levels of phosphatidylcholine lipids containing polyunsaturated fatty acids (PUFAs) provides intriguing mechanistic insight. The chief PUFAs present in these specific phosphatidylcholines were linoleic acid and arachidonic acid, which serve as precursors to eicosanoid signaling molecules that regulate inflammation and insulin sensitivity (31).

Inflammation driven by pro-inflammatory eicosanoids and cytokines exerts detrimental effects on bone by stimulating osteoblast apoptosis and osteoclast differentiation and function. Key cytokines like TNF-alpha and IL-6 promote pathological osteoclastogenesis and bone resorption. Arachidonic acid-derived eicosanoids including prostaglandin E2 also enhance RANKL signaling, fueling excessive osteoclast activity (33, 34). In contrast, anti-inflammatory eicosanoids derived from omega-3 PUFAs such as DHA and EPA help suppress unwarranted osteoclastogenesis and maintain skeletal integrity (32).

Our finding that cereal intake raises phosphatidylcholine levels containing linoleic and arachidonic acid suggests potential modulation of eicosanoid balance favoring bone formation over resorption. Cereal intake may imped pro-inflammatory eicosanoid production through arachidonic acid, while promoting synthesis of lipid mediators like lipoxins and resolvins from omega-3 precursors that protect against cytokine-induced bone loss (35). Increased linoleic acid pools may similarly boost anti-inflammatory metabolites. Beyond effects on eicosanoids, cereal intake could also improve insulin sensitivity through these PUFA-containing phosphatidylcholines, which may help preserve bone mass since insulin resistance promotes osteoporosis (36).

Overall, the identification of PUFA-containing phosphatidylcholines as mediators provides biological plausibility for the protective effect of cereals on skeletal health. The fiber, micronutrients like magnesium and zinc, and phytochemicals present in whole grain cereals likely contribute to increased synthesis of these liposomes or alteration in enzymatic metabolism favoring PUFA incorporation over saturated fats (37). The resultant anti-inflammatory and insulin-sensitizing effects conferred by changes in eicosanoid profiles and membrane dynamics may represent key mechanisms through which cereal intake causally reduces osteoporosis risk.

From a clinical perspective, our findings indicate nutritional strategies emphasizing increased consumption of cereals, fatty fish, and other whole foods abundant in omega-3 PUFAs could help prevent osteoporosis by favorably modulating liposome profiles. Future studies exploring effects of specific dietary interventions on these causal mediating liposomes are warranted. Assessing baseline lipidomics signatures could also help predict individuals likely to benefit the most from particular diet changes. Our results highlight the importance of evaluating both broad dietary patterns and specific bioactive lipid metabolites when developing evidence-based strategies to optimize bone health.

Beyond PUFA-containing phosphatidylcholines, our mediation analysis also uncovered six other liposomes causally linked to elevated osteoporosis odds. These included phosphatidylcholines enriched with saturated fatty acids like palmitic and stearic acid, along with ceramides containing saturated sphingoid bases. Palmitic acid, the primary saturated fat, has been shown to trigger inflammatory cascades and reactive oxygen species that accelerate osteoclastogenesis and bone loss (38). Emerging evidence indicates that saturated fats can negatively impact bone health by interfering with calcium absorption and insulin signaling. Studies have shown that high intake of saturated fats is associated with decreased calcium absorption, which is crucial for bone mineralization (39, 40). Moreover, saturated fats have been linked to impaired insulin signaling, which plays a vital role in bone metabolism. Insulin promotes osteoblast proliferation and differentiation, essential for bone formation, and its impairment can lead to reduced bone accrual (41, 42). These mechanisms underscore the detrimental effects of saturated fats on bone health, highlighting the importance of dietary modifications to prevent osteoporosis.

Meanwhile, ceramides can drive apoptosis of osteoblasts and osteocytes as well as increase osteoclast formation and activity through the stimulation of cytokines like IL-6. Studies have shown that ceramides induce apoptosis in osteoblasts through pathways involving oxidative stress and the activation of pro-apoptotic factors (43, 44). Additionally, ceramides have been implicated in increasing osteoclast formation and activity by stimulating the production of cytokines such as IL-6, which plays a crucial role in bone resorption (45). The accumulation of liposomes containing saturated fatty acids and ceramides likely contributes to the characteristic imbalance favoring bone resorption over formation that underlies osteoporosis pathogenesis (46, 47). Our study nominates this lipidomics signature as a potential biomarker for assessing osteoporosis risk and progression, particularly since liposome levels can be readily measured in blood samples.

From a clinical perspective, these findings suggest nutritional strategies emphasizing diets rich in unsaturated fatty acids from plant sources could help mitigate osteoporosis risk by favorably altering liposome profiles. For instance, adopting Mediterranean-style diets abundant in monounsaturated fats may counter the risks associated with liposomes enriched in saturated fats like ceramides and palmitic acid-containing phosphatidylcholines (48). Future studies exploring effects of specific dietary changes on these causal pro-osteoporotic liposomes are warranted. Patient education on limiting intake of foods high in saturated fats like red meats and full-fat dairy may also help normalize liposome balance to support bone remodeling (47, 49).

Stepping back, a few key overarching themes emerge from our study regarding the power of MR to advance mechanistic comprehension and strengthen causal inference in nutrition research: 1. Integrating multi-omics datasets using MR can unravel the complex interactions between diet, metabolism, and chronic diseases like osteoporosis. By leveraging large GWAS evaluating metabolites and disease outcomes, MR enabled a more rigorous dissection of mediating pathways compared to conventional regression approaches prone to confounding. Findings nominate specific dietary components and bioactive lipids as targets for modification to bolster bone health. 2. Assessing individual foods and distinct varieties rather than solely broad nutrients provided novel insight, as evidenced by the divergent effects of refined white rice vs. fiber-rich cereals. The granularity afforded by food-specific analysis could inform personalized recommendations tailored to individuals' preferences. 3. Beyond elucidating mechanisms, findings pave the way for translational research and clinical applications of causal insights to curb osteoporosis burden. Integration of multi-omics data highlighted promising prognostic indicators and modifiable exposures that could enhance screening and prevention. Collaborating across disciplines will be key to validate and translate discoveries.

Additionally, our study demonstrates the utility of MR for illuminating nuanced mediational pathways. Decomposing the total effect of an exposure like cereal intake into indirect effects operating through mediators like PUFA-containing phosphatidylcholines vs. direct effects independent of the mediator provides biological clarity. The modest 6.9% mediation suggests these liposomes only partially explain cereal intake's protective association. Direct effects may act through additional metabolites or pathways like increased magnesium, phytochemicals, and prebiotics (50). The capacity to disentangle mechanisms makes MR-based mediation analysis a powerful tool for elucidating diet-disease relationships.

In interpreting the results of our MR study, several inherent challenges and potential confounders should be critically considered. Firstly, pleiotropy, where genetic variants influence multiple traits, can potentially confound the results of MR studies. In our analysis, while no evidence of directional pleiotropy was detected using MR-Egger tests, the possibility of non-directional pleiotropy still poses a risk that could distort our causal estimates (51). It is crucial to continue developing and applying robust statistical methods to adequately address this issue and ensure the validity of the MR assumptions.

Population stratification presents another significant challenge. This occurs when differences in allele frequencies coincide with confounders across subpopulations, which can bias the results (52). Our study's findings may not necessarily be generalizable across different ethnic or genetic backgrounds, underscoring the need for replication studies in diverse populations to confirm these findings and enhance their applicability.

Furthermore, our MR analysis primarily focused on a specific subset of dietary components and liposome species, which might not capture the entire spectrum of potential biological interactions and effects. While this focus provided clear insights into certain pathways, it is essential to recognize the limitations and potential biases inherent in this approach. Expanding the analysis to include a broader array of dietary components and integrating additional layers of biological data, such as metabolomics and microbiome data, could provide a more comprehensive understanding of the interactions at play (53).

Selection bias and weak instrument bias are inherent challenges in MR studies. Selection bias can occur if the study sample is not representative of the general population, particularly if the genetic variants used as instruments affect participation probability (54). Weak instrument bias arises when genetic variants do not strongly predict the exposure, potentially leading to attenuated and biased causal estimates. Ensuring that genetic instruments are valid and strong predictors of the exposure is fundamental to mitigating these biases and strengthening causal inference. Future studies should aim to address these challenges through methodological rigor and by expanding the scope of analysis to include more diverse populations (55).

While our study utilized data from Finnish populations, it is crucial to consider the potential for these findings to be applicable to other populations. The genetic background, dietary habits, and environmental factors vary significantly across different populations, which could influence the generalizability of our results. However, the biological mechanisms underlying the associations we observed, such as the impact of specific dietary components on lipid profiles and bone health, are likely to be consistent across different ethnic groups due to the fundamental nature of these physiological processes. Future studies should aim to replicate these findings in diverse populations to confirm their broader applicability. Additionally, understanding population-specific factors that may modify these relationships is essential for developing targeted dietary recommendations for osteoporosis prevention globally (52).

Future research should focus on interventional studies that modify dietary patterns to specifically include or exclude the identified dietary components that influence liposome profiles and osteoporosis risk. Trials that increase the intake of cereals and non-oily fish, while reducing white rice consumption, could provide practical insights into how dietary changes impact osteoporosis outcomes through lipid metabolism. Additionally, a broader lipidomic approach could uncover other lipid components with causal links to osteoporosis, providing a more comprehensive understanding of lipidomic profiles and their biological impacts (56).

## 5 Conclusion

In conclusion, this dual-sample MR study provides novel insights into the causal pathways linking diet, lipid metabolism, and osteoporosis. These insights pave the way for evidence-based dietary recommendations and interventions aimed at improving bone health and reducing the global burden of osteoporosis. By focusing on specific findings and their implications, we aim to translate genetic epidemiological insights into actionable public health strategies.

## Data Availability

The original contributions presented in the study are included in the article/Supplementary material, further inquiries can be directed to the corresponding author.
