# Erratum: Genetic insights into dietary patterns, liposome mediation, and osteoporosis risk: a Mendelian randomization study

**DOI:** 10.3389/fnut.2024.1532886

**Published:** 2024-12-13

**Authors:** Frontiers Production Office

**Affiliations:** Frontiers Media SA, Lausanne, Switzerland

**Keywords:** osteoporosis, dietary habits, liposomes, Mendelian randomization, GWAS, causal inference

Due to a production error, there was an error in affiliation 1. Instead of “Department of Orthopedics, The Third Hospital of Mianyan-Sichuan Mental Health Center, Mianyang, China”, it should read as “Department of Orthopedics, The Third Hospital of Mianyang-Sichuan Mental Health Center, Mianyang, China”.

The publisher apologizes for this mistake.

The original version of this article has been updated.

